
JOURNAL INFORMATION
==============================
NLM Title Abbreviation: ScientificWorldJournal
Journal ID: ScientificWorldJournal
NLM Journal ID: 101131163
Journal ID: TSWJ

The Scientific World Journal

ISSN: 2356-6140
EISSN: 1537-744X
Publisher: Wiley

ARTICLE INFORMATION
==============================
PMCID: PMC5901300
PMID: 17450312
DOI: 10.1100/tsw.2007.96
Article ID: 293764
Article version: 1
Subjects: Clinical Images

Massive Steinstrasse After Treatment of a Staghorn Calculus

Pugliese Jennifer *
Peterson Andrew
Madigan Army Medical Center, Fort Lewis, Tacoma, WA 98431, USA
*Corresponding author.Academic Editor: Daniel Batlle

Publication date: 2007
Electronic publication date: 2007 Apr 9
Volume: 7
First page: 504
Last page: 505
Received 2006 Oct 3; Revised 2007 Feb 27; Accepted 2007 Mar 5
Copyright: Copyright © 2007 Jennifer Pugliese and Andrew C. Peterson.
Copyright year: 2007
License: This is an open access article distributed under the Creative Commons Attribution License, which permits unrestricted use, distribution, and reproduction in any medium, provided the original work is properly cited.
License URL: https://creativecommons.org/licenses/by/3.0/

Keywords: nephrolithiasis, calculus, steinstrasse

==============================
Clinical Image
*TheScientificWorldJOURNAL* (2007) 7, 504–505
ISSN 1537-744X; DOI 10.1100/tsw.2007.96

TheScientificWorldJOURNAL
www.thescientificworld.com

### Massive Steinstrasse After Treatment of a Staghorn Calculus

Jennifer Pugliese* and Andrew Peterson

*Madigan Army Medical Center, Fort Lewis, Tacoma, WA 98431*

*The opinions or assertions contained herein are the private views of the author(s) and are not to be construed as official or as reflecting the views of the Department of Defense*

**E-mail:** llewopjm@yahoo.com; andrew.peterson@amedd.army.mil

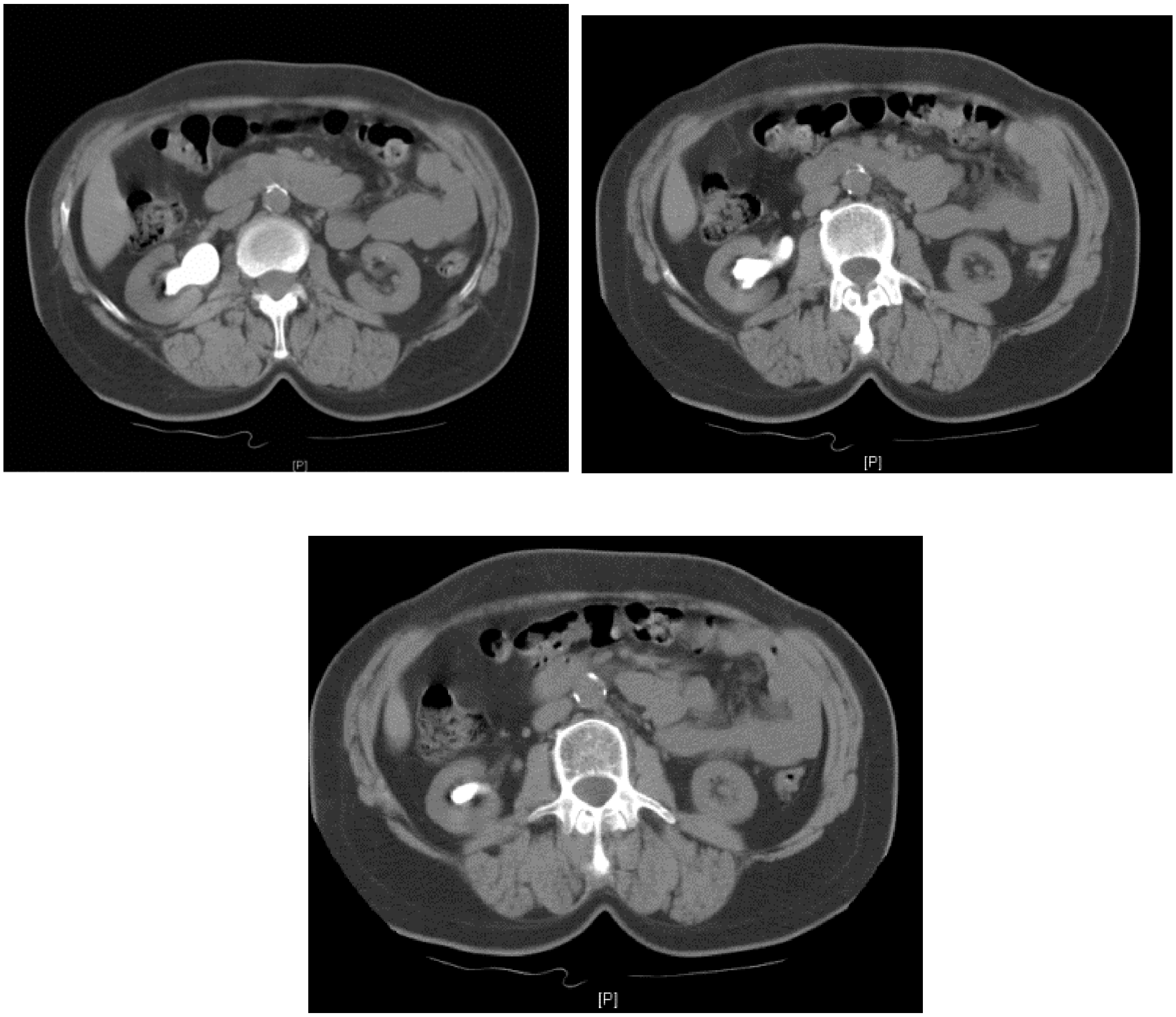

**FIGURE 1.** Computed Tomography with axial images through renal pelvis and ureteropelvic junction demonstrating large preoperative calculus burden.

*Corresponding author.
©2007 with author.
Published by TheScientificWorld; www.thescientificworld.com

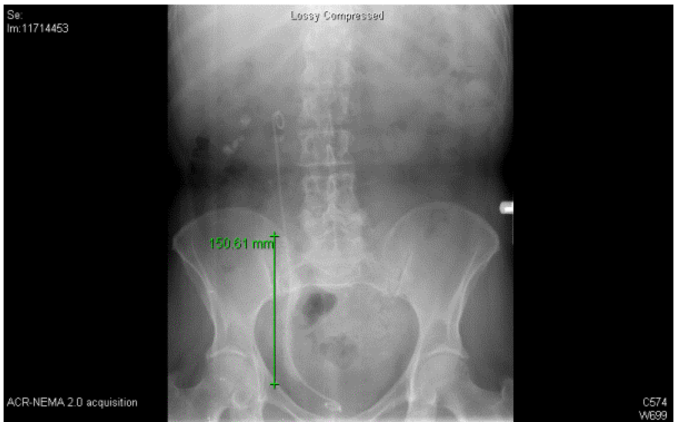

**FIGURE 2.** Plain radiograph demonstrating massive postoperative steinstrasse..

**KEYWORDS:** nephrolithiasis, steinstrasse, calculus

*Received October 3, 2006; Revised February 27, 2007; Accepted March 5, 2007; Published xx, xx, 2007*

A 63-year-old female presented with a symptomatic 2.2 x 3.8 cm right renal stone. After discussion of options for management, she chose minimally invasive treatment. A stent was placed after treatment with right ureteroscopy and laser lithotripsy. She represented with steinstrasse along the length of the stent. This was treated with a combination of ureteroscopy and extracorporal shock wave lithotripsy (ESWL) using 2400 shocks at 24 kilovolts with complete resolution of the steinstrasse.

Steinstrasse may occur after ESWL and is often transient and asymptomatic. Up to one-third of patients may present with silent obstruction and resultant loss of renal function[1]. Steinstrasse usually involves only part of the ureter, and complete ureteral involvement, as in this case, is rare. Abdel-Khalek, et.al. reviewed 2954 patients with renal stones who underwent ESWL. Steinstrasse occurred in 146 cases (4.9%), which resolved spontaneously in 73 (50%)[2]. In a separate review, Weinerth, et.al. showed that large stone burdens (1.7 cm or greater) and bilateral treatements were the largest predisposing factors for the development of steinstrasse[3].

Steinstrasse is a known complication of the treatment of large renal calculi. Since many cases can be associated with asymptomatic obstruction, postoperative imaging is essential for early identification to avoid significant morbidity.

### REFERENCES

1. Madbouly, K. et al. (2002) Risk factors for the formation of a steinstrasse after extracorporeal shock wave lithotripsy: a statistical model. *J Urol.* **167 (3)**, 1239-1242.
2. Abdel-Khalek, M. et al. (2004) Prediction of success rate after extracorporal shock-wave lithotripsy of renal stones—a multivariate analysis model. *Scand J Urol Nephrol.* **38(2),** 161-167.
3. Weinerth, J.L. et al. (1989) Lessons learned in patients with large steinstrasse. *J Urol* **142(6)**, 1425-1427.

**This article should be cited as follows:**

Pugliese, J. and Peterson, A. (2007) Massive steinstrasse after treatment of a staghorn calculus. T*heScientificWorldJOURNAL* **7**, 504–505. DOI 10.1100/tsw.2007.96.
